# *Viridans* streptococcal infective *endocarditis* associated with fixed orthodontic appliance managed surgically by mitral valve plasty: A case report

**DOI:** 10.1097/MD.0000000000011260

**Published:** 2018-07-06

**Authors:** Victoria Birlutiu, Rares Mircea Birlutiu, Victor Sebastian Costache

**Affiliations:** a Lucian Blaga University of Sibiu, Academic Emergency Hospital Sibiu—Infectious Diseases Clinic, Sibiu; b Lucian Blaga University of Sibiu, Spitalul Clinic de Ortopedie-Traumatologie si TBC osteoarticular “Foisor” Bucuresti; c Lucian Blaga University of Sibiu, Faculty of Medicine Sibiu; Department of Cardiovascular and Thoracic Surgery—European Hospital Polisano, Sibiu, Romania.

**Keywords:** braces, case report, fixed orthodontic appliance, infective endocarditis, mitral valve plasty, S*treptococcus viridans*

## Abstract

**Rationale:**

*Streptococcus viridans*, a heterogeneous group of alpha-hemolytic streptococci, is part of the normal flora of the mouth, usually responsible for dental caries (*Streptococcus mutans*, *Streptococcus sanguinis*), and pericoronitis, as well as for subacute infective endocarditis. They are responsible for 40-60% of the endocarditis cases occurring on the normal valves, especially in male patients and over 45 years of age. A change in the bacterial flora of the oral cavity is taking part after orthodontic fixed appliances are introduced into the oral cavity, change that is associated with an increased concentration of the acidogenic bacteria. Bacteraemia is the consequence of oral cavity infections, the association of infective endocarditis with fixed orthodontic appliance, as it has been described by us for the first time, caused by *Abiotrophia defectiva*.

**Patient concerns:**

We present the case of a female Caucasian patient, aged 22 years, who developed infective endocarditis with *Streptococcus viridans* associated with fixed orthodontic appliance, located on the mitral valve, without previous cardiac pathology, and the therapeutic difficulties associated with allergic reactions (to vancomycin, and spironolactone).

**Diagnoses:**

Repetitive haemocultures were positive with *Streptococcus viridans*, while transthoracic echography revealed a severe mitral failure through anteromedial segment of the anterior mitral valve leaf prolapse with eccentric jet to the posterior wall.

**Interventions:**

During hospitalization, the decision to undergo surgical intervention was taken after obtaining negative haemocultures. The patient underwent surgically intervention, and a mitral valve plasty with insertion of neochords was performed.

**Outcomes:**

Intraoperative and subsequently post-discharge transesophageal echography, highlighted normofunctional mitral plasty with a remaining regurgitation grade I-II of IV, with good openness, minor tricuspid regurgitation, and mild pulmonary hypertension.

**Lessons:**

Endocarditis with oral streptococci associated with fixed orthodontic appliance seems to be not so unlikely even in young or without previous cardiac pathology patients, requiring attention in identifying possible pre-existing cardiac conditions like mitral valve prolapse with clinical and echographic monitoring of such cases. Educating and motivating the patient to observe the oral hygiene represent key steps for an optimal oral health during orthodontic treatment. Mechanical tooth cleaning helps maintaining a good oral hygiene during fixed orthodontics and decreasing the oral health risks.

## 1 Introduction

*Streptococcus viridans*, a heterogeneous group of alpha-hemolytic streptococci, is part of the normal flora of the mouth, usually responsible for dental caries (*Streptococcus mutans*, *Streptococcus sanguinis*), and pericoronitis, as well as for subacute infective endocarditis. *S viridans* is responsible for 0.3%–3% of the cases of adult bacterial meningitis, and 1% of those occurring in children. *S viridans* is also responsible for: pulmonary infections (especially in patients with cystic fibrosis), abdominal abscesses, sepsis in immunocompromised patients and neoatal sepsis,^[1–3]^ and osteomyelitis.^[4]^ They are responsible for 40%–60% of the endocarditis cases occurring on the normal valves.^[5,6]^ We present the case of a female Caucasian patient, aged 22 years, who developed infective endocarditis with *S viridans* associated with fixed orthodontic appliance.

## 2 Case report

We present the case of a female Caucasian patient, aged 22 years, which has fixed dental appliance for one year, who was admitted into the Infectious Diseases department for a feverish syndrome associated with migratory joint pain for the last 2 months, gait abnormality, weight loss. She was neurologically, rheumatologically, and imagistically (a lumber magnetic resonance imaging—MRI scan was performed and revealed a normal lumbar spine) investigated. At the time of admission, on physical examination, the following changes were noticed: altered general status, cachexia (BMI of 15.82 kg/m^2^), oxygen saturation 98%, heart rate of 100 beats per minute, systolic murmur in the mitral area grade IV of VI, blood pressure 95 over 60 mm Hg, and a hepatomegaly of 1 cm. Repetitive hemocultures were positive for *S viridans*, while transthoracic echography revealed a severe mitral failure through the anteromedial (A3) segment of the anterior mitral valve leaf (AMVL) prolapse with eccentric jet to the posterior wall. To complete the investigations a transesophageal echocardiography was also performed and certified the diagnosis of mitral valve infective endocarditis (a vegetation of 8 mm was attached to the anteromedial segment of the anterior mitral valve leaf with irregular edges and hypoechogenic aspect). The most important laboratory studies are presented in Table 1.

**Table 1:**
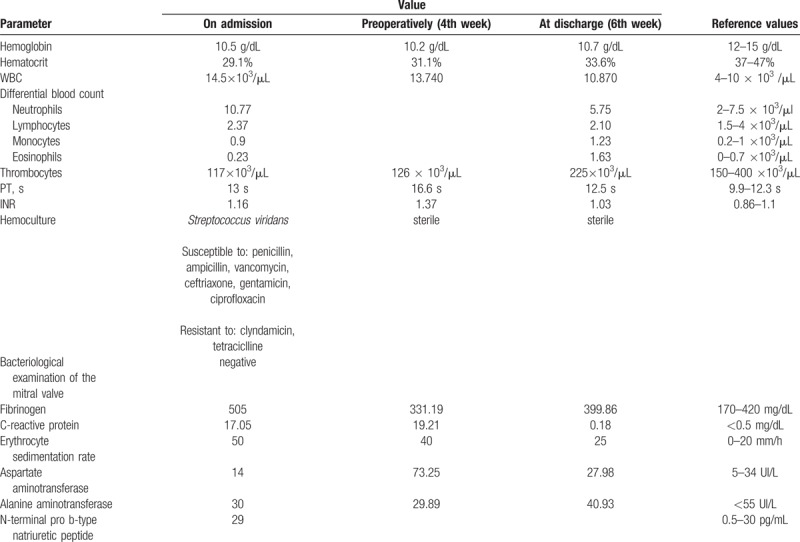
Laboratory studies.

Treatment with Vancomycin and Gentamicin was initiated over the first 2 weeks, in parallel with the extraction of the dental braces, with a slow favourable evolution, the patient becoming afebrile. Subsequently, Ceftriaxone and Vancomycin treatment was continued, under which fever recurred, accompanied by a generalized, intense pruritic erythematous rash (considered as red man syndrome), which led to the cessation of the whole therapy. Antibiotic treatment with ampicillin was initiated, under which the patient became afebrile, allowing the administration of the antibiotic therapy for up to 4 weeks.

After obtaining negative hemocultures, the patient was referred to the Cardiovascular and Thoracic Surgery Department of the European Hospital Polisano for the surgical intervention.

The minimally invasive approach of the mitral valve was made through the v*ideo*-*assisted minithoracotomy.* Upon mitral valve inspection, the presence of small vegetation on the free edge of the anteromedial (A3) segment of the anterior mitral valve leaf and the prolapse of this segment through chordal rupture, has been revealed. The vegetation was excised and 4 GORE-TEX Suture were inserted on the anteromedial (A3) and anteromiddle (A2) segments of the anterior mitral valve leaf. The mitral plasty was completed by the suture of a fully mitral ring Memo 3D ReChord Annuloplasty Ring no.26, with 10 Ethybond Excel 2-0 sutures, fixed with a COR-KNOT device.

Intraoperative and subsequently post-discharge transesophageal echography, highlighted normofunctional mitral plasty with a remaining regurgitation of grade I-II, good openness, minor tricuspid regurgitation, and mild pulmonary hypertension.

## 3 Discussions

*S viridans*, a heterogeneous group of alpha-hemolytic streptococci, is part of the normal flora of the mouth, usually responsible for dental caries (*S mutans*, *S sanguinis*), and pericoronitis, as well as for subacute infective endocarditis. *S viridans* is responsible for 0.3% to 3% of the cases of adult bacterial meningitis, and 1% of those occurring in children. *S viridans* is also responsible for: pulmonary infections (especially in patients with cystic fibrosis), abdominal abscesses, sepsis in immunocompromised patients and neoatal sepsis,^[1–3]^ and osteomyelitis.^[4]^ They are responsible for 40% to 60% of the endocarditis cases occurring on the normal valves.^[5,6]^ The most common association of endocarditis in young people is mitral valve prolapse, especially in male patients and over 45 years of age. Bacteraemia is the consequence of the oral cavity infections. The association of infective endocarditis with fixed orthodontic appliance, has been described by us for the first time in a previous case of infective endocarditis caused by *Abiotrophia defectiva*.^[7]^ Aortic valve endocarditis has also been described as a consequence of a tongue piercing.^[8]^ Exceptionally, the source of endocarditis with *S viridans* may be the adenocarcinoma of the colon.^[9]^

According to the Duke criteria, the diagnosis of infective endocarditis was supported by two major criteria that were met, namely 2 positive hemocultures with *S viridans* and the identification of the vegetation, both echographically and intraoperatively. The use of antibiotic therapy in association with Ceftriaxone and Vancomycin was decided for rapid sterilization of the endocardium prior to surgery, which was scheduled to be performed 4 weeks after treatment. There are studies demonstrating even the efficacy of cephalosporins alone,^[10–13]^ but the monotherapy decision was not taken, given the general condition of the patient and the risk of developing microbial resistance during treatment.

Treatment with Ceftriaxone and Vancomycin was completely replaced due to the generalized rash that the patients developed during the treatment, rash that could have been the cause of an allergic reaction to any of the used antibiotics. The emergence of teicoplanin-induced skin eruption excluded the diagnosis of red man syndrome, suggesting an allergic reaction to glycopeptides. In the attempt to reintroduce spironolactone, the eruption increased, being excluded from treatment. The decision to undergo for a treatment with penicillin was not taken, having in mind the potential risk of penicillin tolerance or even penicillin resistance (up to 13% of the strains being resistant).^[14]^ Although rarely associated with renal failure, septic shock, endocarditis with *S viridans* is more commonly associated with periannular lesions (abscesses, pseudoaneurysms, or fistulas) or heart failure.^[15]^ The risk of death is around 15%.

Surgical intervention was performed on a quasi-emergency basis after four weeks of antibiotic treatment. Classically, mitral endocarditis is approached by median sternotomy, this type of interventions not being practiced by many surgeons due to the difficulty of viewing the mitral valve in acute endocardial lesions when the left atrium is not dilated.^[16]^ Recently, endoscopically minimally invasive mitral surgery has shown its superiority to classical interventions by sternotomy, by decreasing mortality and morbidity.^[17]^

In addition, aesthetic benefits are clearly superior to the endoscopic method, an important aspect in young patients who do not want a scar tissue such as in the case of medial sternotomy. It is also important to perform mitral valve repair in younger female patients, the alternative solution being mechanical valvular prosthesis that would result in the impossibility of completing a pregnancy due to mandatory anticoagulant treatment.

A change in the bacterial flora of the oral cavity is taking part after orthodontic fixed appliances are introduced into the oral cavity, change that is associated with an increased concentration of the acidogenic bacteria, the most important being *S mutans* and *Lactobacilli*. The subgingival bacterial flora increases in number during fixed orthodontics, fixed appliances and the bonding materials are associated with the retention of biofilms. A synergic team work between the orthodontist and dental hygienist should be mandatory and should lead to a decrease of oral health risks associated with the orthodontic treatment. Educating and motivating the patient to observe the oral hygiene and to adhere to the home oral hygiene routine represent key steps for an optimal oral health during orthodontic treatment. Mechanical tooth cleaning provided by a professional dental hygienist help maintaining good oral hygiene during fixed orthodontics and decreasing the oral health risks.^[18,19]^

What is particular to the association of endocarditis with oral streptococci due to fixed orthodontic appliance is the young age of the patients (20–30 years) compared to the usual age of this aetiology, included in the age range of 46–64, as well as the innovative method of repair of the infected mitral valve—mitral valve plasty using video-assisted right minithoracotomy—without previous cardiac pathology. We did not have confirmation of an undiagnosed pre-existing cardiac pathology. Regarding the oral hygiene control of our patient, she presented a good oral hygiene, she attended all the evaluations of the oral hygiene scheduled by her orthodontist and by adhering to the oral hygiene protocol proposed by her dental hygienist.

## 4 Informed consent

Written informed consent was obtained from the patient for publication of this case report and any accompanying images. The study was accepted by the Ethics Committee of the hospital and they encouraged publishing the article. A copy of the written consent is available for review by the Editor-in-Chief of this journal.

## 5 Conclusions

Infective endocarditis with oral streptococci associated with fixed orthodontic appliance seems to be not so unlikely even in young or without previous cardiac pathology patients, requiring attention in identifying possible pre-existing cardiac conditions (like mitral valve prolapse) with clinical and echographic monitoring of such cases. Educating and motivating the patient to observe the oral hygiene represent key steps for an optimal oral health during orthodontic treatment. Mechanical tooth cleaning provided by a professional dental hygienist helps maintaining a good oral hygiene during fixed orthodontics and decreasing the oral health risks.

## Acknowledgments

We express our special thanks to the medical staff from the Infectious Disease Department of the Academic Emergency Hospital from Sibiu, and to the staff from Heart Team—Polisano European Hospital Sibiu.

## Author contributions

All authors contributed equally to this manuscript in terms of acquisition, analysis and interpretation of data, conception and design, drafting the manuscript. All authors read and approved the final manuscript.

**Conceptualization:** Victoria Birlutiu, Rares Mircea Birlutiu, Victor Sebastian Costache.

**Formal analysis:** Victoria Birlutiu, Rares Mircea Birlutiu, Victor Sebastian Costache.

**Investigation:** Victoria Birlutiu, Victor Sebastian Costache.

**Methodology:** Victoria Birlutiu, Victor Sebastian Costache.

**Supervision:** Victoria Birlutiu.

**Validation:** Victoria Birlutiu, Rares Mircea Birlutiu.

**Visualization:** Victoria Birlutiu, Rares Mircea Birlutiu, Victor Sebastian Costache.

**Writing – original draft:** Victoria Birlutiu, Rares Mircea Birlutiu, Victor Sebastian Costache.

**Writing – review & editing:** Rares Mircea Birlutiu.
